# Editorial: Depression, Burnout, and Other Mood Disorders: Interdisciplinary Approaches

**DOI:** 10.3389/fpsyg.2017.00282

**Published:** 2017-03-06

**Authors:** Éric Laurent, Renzo Bianchi, Irvin Sam Schonfeld, Pierre Vandel

**Affiliations:** ^1^Laboratory of Psychology, Université Bourgogne Franche-ComtéBesançon, France; ^2^Maison des Sciences de l'Homme et de l'Environnement, Centre National de la Recherche Scientifique and Université Bourgogne Franche-ComtéBesançon, France; ^3^Institute of Work and Organizational Psychology, Université de NeuchâtelNeuchâtel, Switzerland; ^4^Department of Psychology, The City College of the City University of New YorkNew York, NY, USA; ^5^Laboratory of Neurosciences, Université Bourgogne Franche-ComtéBesançon, France; ^6^Department of Psychiatry, University Hospital, Université de Franche-ComtéBesançon, France

**Keywords:** depressive disorder, mood disorders, multiscale analysis, interdisciplinarity, cognition, evaluation tools, evolutionary approaches, systemic approach

The current *Topic* examines interdisciplinary approaches to mood disorders. The contributions that are gathered in this *Topic* relate concepts from multiple theoretical fields and often rely on mixed-method research. The intention underlying the current *Topic* was to foster connections between the various specialties of psychology and psychiatry that deal with mood disorders. The authors of the present editorial have deployed research efforts for several years in both psychology and psychiatry and have been sensitized to the value of interdisciplinary research through both collaborations (Laurent and Vandel, 2016; Bianchi et al., 2017) and struggle for conceptual parsimony in the field of mood disorders (Bianchi et al., 2017). Research problems in (behavioral) sciences usually involve multiscale structures (Laurent, 2014). The inability to carry out multi-layered investigations often leads to a neglect of either microscale- or macroscale-level determinants of behavior (Laurent and Bianchi, 2016). Unfortunately, this state of affairs can contribute to the development of parallel and poorly-interacting theoretical scaffoldings. For instance, burnout—a construct developed in social and organizational psychology—was introduced in the scientific literature with only limited integration of research on depression—an important category of international psychiatric taxonomies—(Freudenberger, 1974; Maslach and Jackson, 1981; Schonfeld, 1991; Bianchi et al., 2015). As a result, burnout and depression have long been considered distinct entities. Recent research revealed that burnout and depression were actually better conceived of as similar stress-induced processes than as separate conditions (Ahola et al., 2014; Bianchi et al., 2015). Relatedly, in this special issue, Van Dam simultaneously examined burnout and depression, two concepts respectively associated with social psychology and psychiatry, as well as fatigue, a concept traditionally related to somatic medicine. In the framework of cluster analyses, the author showed that subgroup-level analysis is useful to distinguish different levels of burnout based on depression, anxiety and fatigue symptoms, with depression being the strongest predictor of group membership. Interdisciplinary approaches to mood disorders are also key to the understanding of mental health dynamics following real-life events in complex situations. Shi et al. have coordinated the psychiatry of depression, a psychosocial approach to living conditions, and personality psychology. Their longitudinal research included data concerning depressive symptoms in adolescent survivors after the 2008 Wenchuan earthquake. They have shown that survivor depressive symptoms are related to gender, dispositional resilience and social factors such as social support. Their complexity-oriented approach informs us about the temporal dynamics of the different influences on depression. Complexity was also featured in Dumitrescu's paper, which describes links between periodontal disease and depression. In an original manner, the author has examined reciprocal influences between the bacterially-mediated inflammatory disease and depression. This type of approach helps narrow the gap between what has been usually regarded as a somatic disease with microscale-level determinism and what has often been categorized as a “mental” disorder. Two other papers (Brockmeyer et al.; Carvalho et al.) highlight the interplay among cognition, behavior, and depressive disorders. Brockmeyer et al. have investigated the role of depression in self-focused attention as a function of the “affective context” (i.e., positive vs. negative valence of recalled events). Linguistic markers (i.e., use of first-person singular pronouns in natural language) were used to characterize maladaptive self-focus in some contexts. Carvalho et al. have reviewed the literature on saccadic eye movements in unipolar depression (UD) and bipolar disorder (BD). They have shown that oculomotor behavior can be differently altered in both disorders. Thus, they have also discussed the discriminative potential of eye movement paradigms in the differential diagnosis between UD and BD. Finally, Clarke et al. assessed the feelings of (arrested) anger and (arrested) escape leading to a perception of entrapment in patients presenting with recent experiences of self-harm. The authors have built on evolutionary theories of psychopathology. They have examined psychopathology (e.g., depressive disorders) and suicide within evolutionary and behavioral approaches, stressing the role of arrested (aroused) defenses in psychological disorders and self-harm. They have also offered the reader a tool to assess arrested fight and flight (escape, anger) and self-hurting and risk behavior history.

The current issue brings together various perspectives on mood disorders and psychopathology, which all have in common (i) an interdisciplinary perspective and a complexity-oriented approach to their research object and (ii) an interest in both basic problems in science and potential far-reaching consequences of research applications.

In our view, “interdisciplinary research” and “mixed methods” are not just fashionable words; they represent a route to tackle complexity in science.

## Author contributions

ÉL wrote the initial draft of the document. ÉL, RB, IS, and PV contributed to review the manuscript.

### Conflict of interest statement

The authors declare that the research was conducted in the absence of any commercial or financial relationships that could be construed as a potential conflict of interest.
